# Enhancer of Zeste Homolog 2 (EZH2) Is a Marker of High-Grade Neuroendocrine Neoplasia in Gastroenteropancreatic and Pulmonary Tract and Predicts Poor Prognosis

**DOI:** 10.3390/cancers14122828

**Published:** 2022-06-08

**Authors:** Sebastian C. B. Bremer, Gabi Bittner, Omar Elakad, Helen Dinter, Jochen Gaedcke, Alexander O. König, Ahmad Amanzada, Volker Ellenrieder, Alexander Freiherr von Hammerstein-Equord, Philipp Ströbel, Hanibal Bohnenberger

**Affiliations:** 1 Clinic for Gastroenterology, Gastrointestinal Oncology and Endocrinology, University Medical Center Goettingen, Georg-August-University, 37075 Goettingen, Germany; alexander.koenig@med.uni-goettingen.de (A.O.K.); ahmad.amanzada@med.uni-goettingen.de (A.A.); volker.ellenrieder@med.uni-goettingen.de (V.E.); 2 Institute of Pathology, University Medical Center Goettingen, Georg-August-University, 37075 Goettingen, Germany; bittnergabi@outlook.de (G.B.); omar.elakad@med.uni-goettingen.de (O.E.); dinterhelen@gmail.com (H.D.); philipp.stroebel@med.uni-goettingen.de (P.S.); hanibal.bohnenberger@med.uni-goettingen.de (H.B.); 3 Clinic for General, Visceral and Pediatric Surgery, University Medical Center Goettingen, Georg-August-University, 37075 Goettingen, Germany; jochen.gaedcke@med.uni-goettingen.de; 4 Clinic for Cardiac, Thoracic and Vascular Surgery, University Medical Center Goettingen, Georg-August-University, 37075 Goettingen, Germany; alexander.hammerstein@med.uni-goettingen.de

**Keywords:** neuroendocrine neoplasia, EZH2, survival

## Abstract

**Simple Summary:**

Neuroendocrine neoplasms most frequently arise in the gastroenteropancreatic and pulmonary tract and show an increasing incidence and prevalence. The prognosis and treatment depend on tumor proliferation and clinical behavior. Highly proliferating grade 3 neoplasms especially, show a wildly divergent therapy response and prognosis. In particular, it is crucial to securely separate the more indolent G3 tumors from the more aggressive carcinomas. Currently, this distinction is based on a combination of clinical, morphologic, immunohistochemical, and molecular biomarkers. However, none of these markers allow for a reliable distinction, and additional markers are needed. EZH2 has attracted increasing interest in different tumor entities. We aimed to analyze the expression of EZH2 in different neuroendocrine neoplasms and to correlate the expression with clinical parameters and survival. We demonstrate that EZH2 is nearly exclusively expressed in highly proliferative neoplasms and is a robust biomarker for identifying aggressive G3 tumors with poor prognosis.

**Abstract:**

Tumor grading is a robust prognostic predictor in patients with neuroendocrine neoplasms (NEN) and guides therapy, especially in tumors with high proliferation. NEN can be separated into well-differentiated and poorly differentiated types. The more aggressive NEN have been further separated into neuroendocrine tumors (NET G3) with a better prognosis and neuroendocrine carcinomas (NEC) with a worse prognosis. Despite this distinction’s tremendous clinical and therapeutic relevance, optimal diagnostic biomarkers are still lacking. In this study, we analyzed the protein expression and prognostic impact of Enhancer of Zeste Homolog 2 (EZH2) by immunohistochemistry in 219 tissue samples of gastroenteropancreatic (GEP-NEN) and pulmonary NEN (P-NEN). EZH2 was almost exclusively expressed in NEN with a proliferation rate above 20% (G3), while all low-grade tumors were nearly negative. Among high-grade NEN, 65% showed high and 35% low expression of EZH2. In this group, the high expression of EZH2 was significantly associated with poor overall survival and NEC histology. Interestingly, EZH2 seems to act independently of Polycomb Repressive Complex 2 (PRC2) in NEN. In conclusion, we propose EZH2 as a robust biomarker for distinguishing between NET G3 and NEC among gastroenteropancreatic and pulmonary NEN.

## 1. Introduction

Neuroendocrine neoplasms (NEN) can arise in many different organs but are most frequently found in the gastroenteropancreatic (GEP-NEN) and pulmonary (P-NEN) tract [1]. The incidence and prevalence of NEN are continuously rising [1]. According to the WHO and ENETS classification, NEN can be separated into well-differentiated tumors (NET) and poorly differentiated carcinomas (NEC) [2,3,4].

P-NEN are classified into low-grade typical carcinoids (TC, G1), intermediate-grade atypical carcinoids (AC, G2), and high-grade large cell neuroendocrine carcinomas (LCNEC, G3), whereas GEP-NEN are classified in NET G1, G2, G3, and NEC [5]. The grading of NEN depends on the proliferation measured as mitotic count in P-NEN or Ki67 index in GEP-NEN, respectively. In P-NEN, a mitotic count of 0–2 mitoses per 2 mm^2^ is defined as typical carcinoid (G1), 2–10 as atypical carcinoid (G2), and >10 as large cell neuroendocrine carcinoma (G3) [6]. In GEP-NEN, a Ki67 rate of <3% is defined as G1, 3–20% as G2, and >20% as G3 [4].

The prognosis of NEN correlates strongly with the tumor grade that is currently mainly based on proliferation [7]. However, some highly proliferative tumors are associated with slow progression and better prognosis, while others pursue an aggressive course [7]. Therefore, in GEP-NEN, the current nomenclature has introduced the subgroup of NET G3 with a prognosis intermediate between NET G1/G2 and NEC [7]. While NEC may require aggressive chemotherapy in addition to oncological resection, the standard therapy of NET G3 is currently still unclear and depends much more on their clinical presentation and course [8]. Accordingly, it is crucial to securely separate the more indolent NET G3 from aggressive NEC [7]. At the moment, this distinction is based on a combination of several clinical, morphologic, immunohistochemical, and molecular biomarkers. These histological markers include Chromogranin A (CgA), DAXX/ATRX, RB, SSTR2A, and p53 [9].

Ki67 is expressed during all cell cycle phases but not in the G0 phase [10]. Therefore, Ki67 has established itself as a cell proliferation marker and is crucial for grading neuroendocrine neoplasia, especially GEP-NEN [11]. However, the Ki67 index alone is not sufficient for distinguishing between NEN-G3 and NEC; the morphology and expression of various markers are also required [12]. Chromogranin A is a glycoprotein secreted by neuroendocrine cells, and its expression is usually maintained in well-differentiated NETs but frequently lost in NECs [13,14,15]. ATRX/DAXX is involved in chromatin remodeling and telomere length regulation [16]. DAXX (death domain associated protein) is a specific histone chaperone, and its targets can be controlled by ATRX [16]. The loss of DAXX/ATRX is observed in about 40% of well-differentiated NETs, while it is maintained in NECs [13,17]. P53 mutations can be found in up to 76% of NECs [18]. Although the sensitivity of immunohistochemistry in the screening for p53 mutations is only moderate, most cases in daily practice will primarily be assessed by immunohistochemistry, and p53 is an important marker for diagnosing NEC [18]. However, none of these markers allow a reliable distinction on their own, and due to the significant clinical consequences, additional markers are needed to differentiate NET G3 and NEC more reliably.

In recent years, one marker that has attracted increasing interest in various cancer entities is Enhancer of Zeste Homolog 2 (EZH2) [19]. We recently demonstrated that immunohistochemical assessment of EZH2 is very useful for differentiating thymic NET G3 from NECs [20]. EZH2 is the catalytic subdomain of the Polycomb Repressor Complex 2 (PRC2). The other core subunits of PRC2 are Suppressor of Zeste Homolog-12 (SUZ12), Embryonic Ectoderm Development (EED), and Enhancer of Zeste Homolog 1 (EZH1) [21]. PRC2 mediates primarily trimethylation of histone 3 lysine 27 (H3K27me3) via its SET (Su[var]3–9, Enhancer of Zeste, and Trithorax) domain [22,23]. This trimethylation leads to local heterochromatin-mediated gene-silencing [24]. Nevertheless, PRC2-independent functions of EZH2, e.g., the modification of protein activity by methylation or direct binding, have also been described in different cancer entities [25,26,27,28,29,30]. EZH2-mediated gene-silencing and other epigenetic mechanisms in cancer development and progression have gained increasing interest and offer new treatment options [19,31,32]. In most cancer entities (e.g., pancreatic cancer, breast cancer, prostate cancer, lung cancer), EZH2 is clearly associated with pro-tumorigenic and tumor-progressive characteristics [33,34,35,36,37,38,39]. In colorectal cancer, in contrast, EZH2 expression appears to be associated with a more favorable prognosis [40].

This study aimed to assess EZH2 expression in different types of GEP-NEN and P-NEN and to analyze whether EZH2 can help in the distinction between NET G3 and NEC.

## 2. Materials and Methods

### 2.1. Ethics

This project was approved by the ethics committee of the University Medical Center Göttingen (#10/11/20). Informed consent was obtained from all patients. All procedures were conducted in accordance with the declaration of Helsinki and institutional, state, and federal guidelines.

### 2.2. Patient Enrollment for Immunohistochemistry

We examined 219 tissue specimens of gastroenteropancreatic (GEP-NEN) and pulmonary NEN (P-NEN) that were collected during surgical resection. Every tumor sample was from a separate patient. Additionally, we collected data on these patients’ cancer stage and survival. All samples were formalin-fixed and paraffin-embedded (FFPE) and diagnosed at the Institute of Pathology at the University Medical Center Göttingen.

### 2.3. Immunohistochemical Staining

Tissue samples were assembled in tissue microarrays before immunostaining. Immunohistochemical stainings were performed as described previously [41]. In short, 2-μm tissue sections were incubated in EnVision Flex Target Retrieval Solution, pH low or high (Dako, Glostrup, Denmark). After this procedure, the probes were incubated with primary antibodies against EZH2 (Leica, Wetzlar, Germany, EZH2-L-CE, 1:100, high), EZH1 (Merck, Darmstadt, Germany, #ABE281, 1:1000, low), SUZ12 (Sigma, St. Louis, MO, USA, HPA057436, 1:100, low), EED (Sigma, HPA061140, 1:100, high), H3K27me3 (Cell signaling, Danvers, MA, USA, 9733S, 1:500, high), Ki-67 (Dako/Agilent, Santa Clara, CA, USA, clone MIB-1, 1:200, low), DAXX (Sigma, HPA008736, 1:200, low), ATRX (ThermoFisher, Waltham, MA, USA, clone CLO 537, 1:100, low), TP53 (Agilent, Santa Clara, CA, USA, GAG16, RTU, high), RB (Sigma, rabbit polyclonal, 1:100, high), SSTR2A (Zytomed Systems, Berlin, Germany, rabbit polyclonal, 1:100, high) or Chromogranin A (Cell Marque, Rocklin, CA, USA, LKH210, RTU, high) at room temperature for 20 min. Polymeric secondary antibodies coupled to HRPO peroxidase (EnVision Flex+, Dako) and DAB (Dako) were applied for visualization of the sites of immunoprecipitations. After counterstaining with Meyer’s hematoxylin, the stainings were analyzed by light microscopy. Staining intensity was evaluated by using the H-score (1 × (%cells with weak intensity) + 2 × (%cells with intermediate intensity) + 3 × (%cells with strong intensity)) for every sample leading to a range of 0–300.

### 2.4. Statistics

Data are displayed as box and whisker plot in the style of Tukey and analyzed with Kruskal–Wallis test followed by Dunn’s multiple comparisons test or as survival curves calculated with Kaplan–Meier method and analyzed with log-rank test. In order to test for normal distribution of the data a Shapiro–Wilk test was performed.

Differences between two groups were analyzed with two-sided chi-square test. For the analysis and plot construction, we used GraphPad Prism 9 (Graphpad Software, San Diego, CA, USA, Version 9.2.0).

Multiple regression analysis model was performed with IBM SPSS Statistics version 27.0.0.0. Statistical significance was defined as *p* < 0.05 (confidence level 95%). Overall survival (OS) was defined as the time from first diagnosis until death by any cause.

## 3. Results

### 3.1. Patient Characteristics

A total of 219 patients (113 male, 106 female) were included in this study. Of these, 126 patients had gastroenteropancreatic NEN (GEP-NEN), while 93 patients were diagnosed with pulmonary NEN (P-NEN). All patient characteristics are summarized in Table 1 and further detailed in supplementary Table S1 (NEN G1/2) and Table S2 (NEN G3).

**Figure 1:**
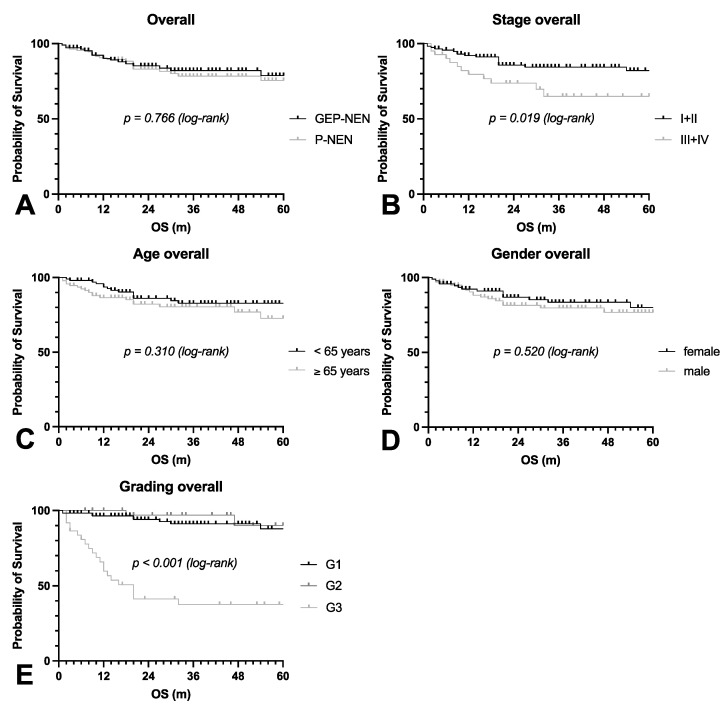
Overall survival based on clinical characteristics. The overall survival (OS) of all enrolled patients with GEP-NEN or P-NEN was calculated (**A**). Additionally, the OS with respect to the UICC stage (**B**), age (**C**), gender (**D**), and grading (**E**) was calculated. GEP: gastroenteropancreatic, NEN: neuroendocrine neoplasia, OS: overall survival, P: pulmonary.

The 5-year overall survival of GEP-NEN and P-NEN was identical (*p* = 0.766), even though GEP-NEN were diagnosed more often in higher tumor stages (Figure 1A). Survival correlated with the UICC tumor stage (*p* = 0.019) (Figure 1B). The median age was 65 and 62 years in GEP-NEN and P-NEN with a predominance of males in GEP-NEN and a predominance of females in P-NEN. Survival was unrelated to age (*p* = 0.310) or gender (*p* = 0.520) (Figure 1C,D).

The most decisive prognostic factor in our collection was pathological grading (*p* < 0.001). Survival was similar in patients with G1 or G2 tumors and significantly worse in patients with G3 tumors (Figure 1E).

**Figure 2:**
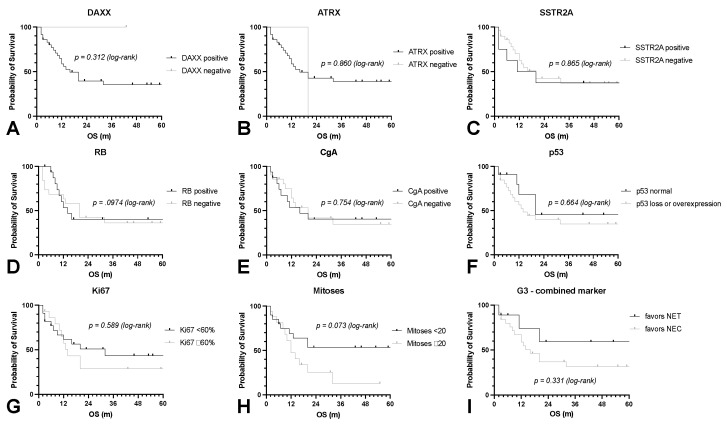
Overall survival in relation to histological markers. The overall survival (OS) of all enrolled patients with NEN G3 was calculated with respect to the expression or intensity of DAXX (**A**), ATRX (**B**), SSTR2A (**C**), RB (**D**), Chromogranin A (CgA, (**E**)), p53 (**F**), Ki67 (**G**) and mitoses (**H**). Two groups were distinguished by taking all markers together: favors NET G3 and favors NEC (**I**). GEP: gastroenteropancreatic, NEC: neuroendocrine carcinoma, NEN: neuroendocrine neoplasia, NET: neuroendocrine tumor, OS: overall survival, P: pulmonary.

Currently, the discrimination between NET G3 and NEC relies on a combination of several morphologic and immunohistochemical markers. Morphologically, NET G3 display carcinoid morphology with trabecular growth patterns, delicate vasculature, and pepper-and-salt chromatin, while NEC typically grow in solid or sheet-like patterns, show extensive necrotic areas, and display frank cytological atypia. Furthermore, NEC have higher Ki67 and mitotic indices on average, and a very high number of mitoses (>20 mitosis/10 high power fields) favors the diagnosis of NEC. However, a considerable overlap between the two groups limits the diagnostic value in individual cases. Further immunohistochemical markers that favor the diagnosis of NET G3 are the conserved expression of Chromogranin A and SSTR2A and a physiological expression of p53. In a subset of NET G3, mutations of ATRX or DAXX can lead to a loss of expression as another indicative biomarker. On the other hand, the diagnosis of NEC is favored by abnormal p53 expression (overexpression or loss) and the loss of Chromogranin A, SSTR2A, or RB expression. Interestingly, none of the described markers alone were associated with significantly different overall survival (Figure 2A–H). Taking together all of the described morphological and immunohistochemical markers, we were able to tentatively assign 25 of the NEN G3 cases to the NEC group (favors NEC) and 9 to the NET G3 group (favors NET). Surprisingly, the overall survival was not significantly different between these two groups (*p* = 0.331, Figure 2I).

Two cases with carcinoma morphology and p53 overexpression showed strong expression of Chromogranin A and SSTR2A. One other tumor had a carcinoid morphology but overexpression of p53, a loss of SSTR2A, and a high mitotic count (28/10 HPF). To our understanding, such cases currently cannot be assigned to one of the two groups.

We also analyzed our cohort separately for localization (GEP-NEN vs. P-NEN). The OS with respect to the tumor stage showed no difference in GEP-NEN (*p* = 0.119) but showed a difference in P-NEN (*p* = 0.001). Age and gender showed no significant difference in both groups. G3 tumors showed significantly worse survival in both groups (*p* < 0.001 in GEP-NEN and P-NEN). Data are shown in supplementary Figure S1.

### 3.2. EZH2 Expression Strongly Predicts Patient’s Survival in NEN G3

**Figure 3:**
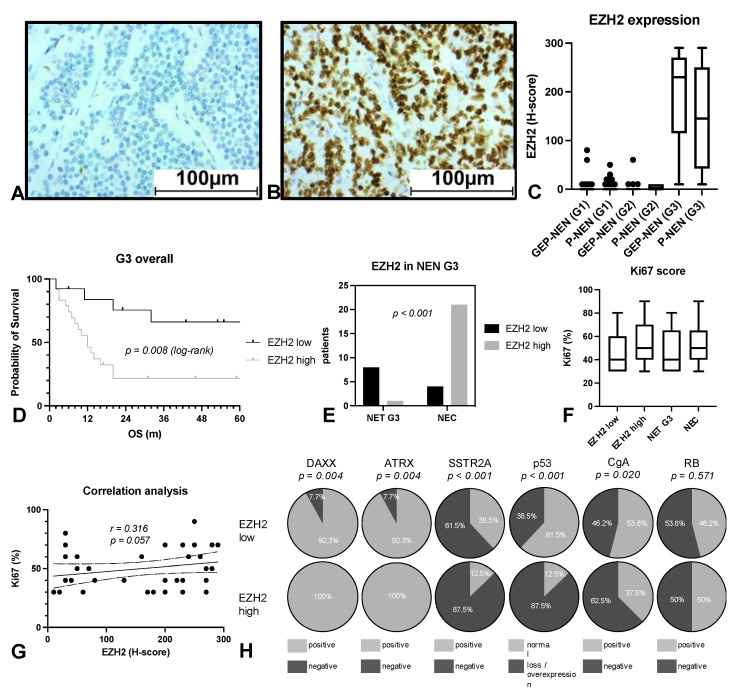
EZH2 expression in NEN. EZH2 staining was performed in all specimens. (**A**) shows an example of very low EZH2 expression, while (**B**) shows very high expression. EZH2 expression was quantified by using the H-score and correlated with grading and entity (**C**). Overall survival (OS) in relation to EZH2 expression (**D**). The distribution of EZH2 in NET G3 and NEC samples was analyzed and calculated (**E**). The distribution of Ki67 in tumors with low or strong EZH2 expression and in NET G3 and NEC and the direct correlation of EZH2 and Ki67 were analyzed (**F**,**G**). Correlation of conventional marker of NEN G3 and EZH2 expression (**H**). GEP: gastroenteropancreatic, NEN: neuroendocrine neoplasia, OS: overall survival, P: pulmonary.

Next, we analyzed the expression of EZH2 in all samples by immunohistochemistry and found a very low expression in all G1 and G2 tumors. Most cases were completely negative, and only a very few samples showed a weak expression. In stark contrast, G3 tumors (both GEP-NEN and P-NEN) showed a strong expression and significantly higher mean values as a group (Figure 3A–C). Interestingly, there was a marked dichotomy even among G3 tumors: most G3 tumors showed either very strong or very weak EZH2 expression. Therefore, it was easy to define and compare tumors with high and low EZH2 expression. We defined an H-score of 100 as the cutoff based on our finding that no low-grade NEN had a score > 100. Among 17 GEP-NEN G3, n = 4 showed low and n = 13 high expression of EZH2. Among 20 P-NEN-G3, n = 9 showed low and n = 11 high expression of EZH2. High EZH2 expression was significantly and strongly associated with worse overall survival in NEN G3, with a median survival of 12 months in the EZH2 high group vs. 81 months in the EZH2 low group (HR 3.377 (1.384–8.240, *p* = 0.008)) (Figure 3D).

Furthermore, EZH2 expression was strongly associated with the aforementioned distribution of NET G3 and NEC in our cohort. Using EZH2 staining with a cutoff of H-score = 100, we were able to confirm the classification of NEN G3 cases as either NET G3 of NEC in 29 of the 34 cases (concordance of 85.3%, *p* < 0.001) (Figure 3E). Two cases that had been unequivocally assigned as NEC due to their carcinoma morphology as well as p53 overexpression and loss of expression of RB, SSTR2A, and Chromogranin A had an EZH2 H-score of 50 and 80, respectively. Interestingly, one patient had an OS of 32 months, and the other is still alive after 55 months. In another two cases with carcinoid morphology and an EZH2 H-Score of 50 and 70, a diagnosis of NEC had initially been favored because of p53 overexpression and the loss of Chromogranin A and SSTR2A. However, the patients were still alive after 133 and 157 months of follow-up.

In contrast, one tumor with carcinoid morphology, preserved expression of Chromogranin A, and physiological expression of p53 showed an EZH2 H-score of 270. Although the case had initially been assigned to the NET G3 group, this tumor had had an exceptionally high mitotic count of 42 in 10 HPFs. Unfortunately, the patient was lost for follow-up.

One of the three described cases that had initially been deemed as not classifiable had an EZH2 H-score of 40, favoring classification as NET G3. The patient was still alive after 93 months of follow-up. The other two cases had an EZH2 H-score of 250 and 280, favoring their classification as NEC. The two patients deceased 6 and 10 months after diagnosis.

We further analyzed whether EZH2 correlated with the Ki67 but found no significant association (r = 0.316, *p* = 0.057, Figure 3F,G).

In addition, the expression of the histological markers DAXX (*p* = 0.004), ATRX (*p* = 0.004), SSTR2A (*p* < 0.001), p53 (*p* < 0.001), and CgA (*p* = 0.020) that are used to distinguish between NET G3 and NEC in GEP-NEN also differed significantly among G3 tumors with high and low EZH2 expression (Figure 3H). Only RB showed no significant difference (*p* = 0.571).

On univariate analysis, EZH2 expression was strongly associated with higher tumor grade, pT status, and tumor stage. Furthermore, strong EZH2 expression was more frequent in male patients. No significant correlation with age or localization was found (Table 2). Multivariate analysis confirmed that grading, pT status, and staging, but not gender, were significantly associated with EZH2 expression (Table 3).

In P-NEN, high EZH2 expression was significantly associated with worse OS (*p* < 0.001). In GEP-NEN, there was a clear trend towards shorter survival in the EZH2 high group that did not reach statistical significance, possibly because the EZH2 low group was very small (n = 4). H3K27me3 expression was not correlated with OS in both groups (Supplementary Figure S2). Further subanalysis of the exact tumor localization (pancreatic vs. intestinal vs. pulmonary NEN) revealed no site specific differences in OS or EZH2 expression. EZH2 expression was significantly higher in G3 tumors independent of their localization (Supplementary Figure S3 and Table S3).

### 3.3. EZH2 Influences Survival on NEN Patients Independently of PRC2

**Figure 4:**
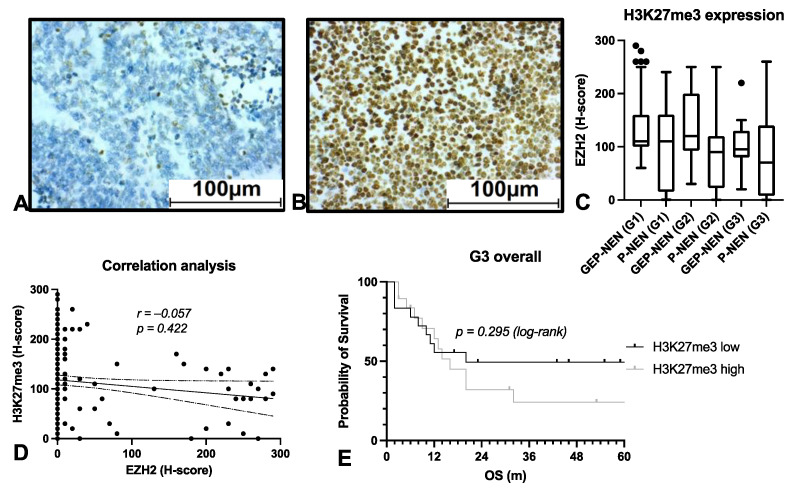
H3K27me3 expression in NEN. H3K27me3 staining was performed in all specimens. (**A**) shows an example of very low H3K27me3 expression, while (**B**) shows very high expression. H3K27me3 expression was quantified using the H-score and correlated with grading and entity (**C**). We performed a correlation analysis of H3K27me3 and EZH2 (**D**). Overall survival (OS) in correlation with H3K27me3 expression (**E**). GEP: gastroenteropancreatic, NEN: neuroendocrine neoplasia, OS: overall survival, P: pulmonary.

In order to obtain a further insight into the function of EZH2 in NEN, we analyzed immunohistochemical stainings of trimethylated H3K27 in all samples (Figure 4A,B). Surprisingly, the staining intensity of H3K27me3 was similar across all grades and entities (Figure 4C). Thus, there was no significant correlation with EZH2 expression (r = 0.057, *p* = 0.422) (Figure 4D) or overall survival (*p* = 0.295) (Figure 4E).

**Figure 5:**
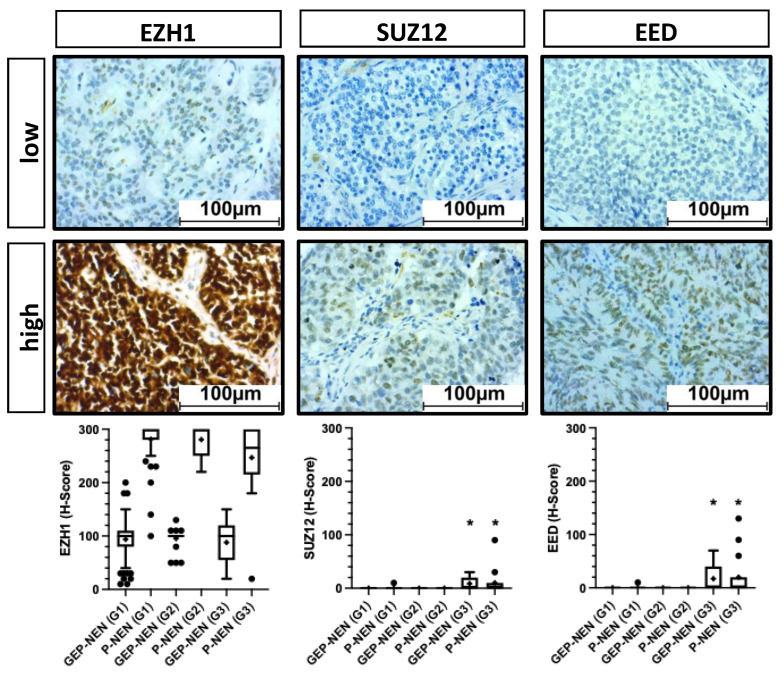
Expression of PRC2 members. Other members of the Polycomb Repressor Complex 2 (PRC2) were stained and quantified using the H-score. Expression was correlated with grading and entity (bottom line). The left column shows the results for EZH1, the middle column for SUZ12, and the right column for EED. EED: Embryonic Ectoderm Development, EZH1: Enhancer of Zeste Homolog 1, GEP: gastroenteropancreatic, NEN: neuroendocrine neoplasia, OS: overall survival, P: pulmonary, SUZ12: Suppressor of Zeste 12, *: significant compared to G1 and G2.

In addition, we examined the expression of other core members of the Polycomb Repressive Complex 2 (PRC2). Interestingly, the mean H-Score for EZH1 was significantly higher in P-NEN compared to GEP-NEN but similar for all tumor grades, while SUZ12 and EED were barely expressed across all grades and entities (Figure 5). Although SUZ12 and EED expression was significantly higher in G3 tumors, their absolute expression was very weak.

Collectively, we did not find evidence that the strong prognostic effect of EZH2 in NEN depended on the expression level of other Polycomb Repressive Complex 2 members.

## 4. Discussion

This work aimed to establish markers that assist pathologists in the clinically relevant separation of NET G3 tumors and NEC. NET G3 tumors were first described in the gastroenteropancreatic system, but have subsequently also been recognized in other organs, including the thymus [20,42]. In the lung, the same concept seems to apply, and these tumors are currently called carcinoid tumors with elevated mitotic count/high proliferation index [43,44]. However, the number of published cases is still low and needs to be increased for a better characterization of these tumors. This study analyzed a cohort of 219 GEP-NEN and P-NEN, including 37 gastroenteropancreatic and pulmonary NEN-G3 tumors, for their expression of EZH2 and PRC2 core members.

Histopathological tumor grading was the factor with the greatest impact on prognosis in our collection. In line with previous reports, overall survival was similar in patients with G1 or G2 tumors and was significantly worse in patients with G3 tumors [45]. In our cohort and other series, age and gender had no significant influence on overall survival [46]. Man et al. analyzed an epidemiologic database with more than 70,000 patients and found a significant difference in the survival of localized and regional NENs, albeit weaker than the impact of grading [45]. We observed similar survival in patients with GEP-NEN and P-NEN despite significant differences in the distribution of UICC stages in the two cohorts. This finding underscores the importance of grading in the survival prediction of NEN, irrespective of their anatomic localization.

In 126 GEP-NEN and 93 P-NEN cases, 17 (13.5%) and 20 (21.5%) NEN-G3 cases, respectively, were included. As described before, the discrimination of NET G3 and NEC currently still relies on several morphologic and immunohistochemical criteria [7,9,20]. For further classification of our collection, we evaluated morphology, mitotic count, Ki67 index, and the expression of p53, RB, Chromogranin A (CgA), SSTR2A, DAXX, and ATRX. Interestingly, none of the markers alone could predict the patients’ prognosis. The best single marker for differentiation of prognosis was a high mitotic count, defined as more than 20 mitoses in 10 high power fields. When taking together all of the described morphological and immunohistochemical markers, we were able to tentatively assign 25 of the NEN G3 cases as NEC and 9 as NET-G3 (5 of 17 (29.4%) in GEP-NEN and 4 of 20 (20%) in P-NEN). The percentages are comparable to the results of the PRONET prospective study [47]. In our series, a trend towards shorter overall survival of NEC cases was observed but did not reach statistical significance (*p* = 0.331), underlining the difficult subclassification of NEN G3 cases.

Enhancer of Zeste Homolog 2 (EZH2) has attracted increasing interest in recent years, and specific molecular inhibitors are currently being tested in various cancer entities. The FDA has already approved their clinical use for refractory follicular lymphoma and epithelioid sarcoma. We recently demonstrated that EZH2 expression helps differentiate thymic NET G3 from NECs [20]. Furthermore, a correlation between EZH2 expression and elevated proliferation and dysregulated p53 expression has been described in intestinal NEN, and a significantly stronger expression of EHZ2 has been reported in high-grade compared to low-grade NEN of the lung [48,49]. The development of neuroendocrine prostate cancer seems to be mediated by EZH2 [50,51]. Furthermore, several preclinical studies have demonstrated that direct or indirect targeting of EZH2 is a potential new therapeutic strategy for NEN of the pancreas and small intestine [32,52,53].

In our study, strong expression of Enhancer of Zeste Homolog 2 (EZH2) was virtually absent in well-differentiated G1 and G2 NEN, while G3 tumors showed a dichotomous expression with either very weak or very strong expression. In NEN G3, EZH2 overexpression alone could recapitulate the subclassification into NET G3 and NEC and was significantly and strongly associated with poor prognosis. Patients with low EZH2 expression presented with only slightly worse overall survival than patients with NEN G1/2 tumors. Multivariate analysis revealed that grading was most strongly associated with high EZH2 expression, followed by pT status and UICC stage, further underlining the aggressive behavior of EZH2 positive NENs.

Interestingly, EZH2 predicted overall survival more precisely than any other tested markers (CgA, DAXX/ATRX, RB, SSTR2A, mitoses, Ki67, or p53) alone and even better than all of these markers in combination. Among the 37 NEN G3 cases, three had been deemed unclassifiable by current criteria. In another five cases, EZH2 expression did not fit to their tentative classification as either NET G3 or NEC by current criteria. However, in seven of these eight equivocal cases, low and high EZH2 expression was able to predict the survival of these patients correctly. In one of these cases (a tumor with exceptionally high mitotic count), the patient was lost for follow-up. These observations based on a small numbers of cases certainly need further investigations to confirm the exact role of EZH2 but underline its strong prognostic power in NEN G3.

Finally, we aimed to understand further the mechanisms underlying the critical role of EZH2 in high-grade NENs. Classically, EZH2 acts as the catalytic subdomain of the Polycomb Repressor Complex 2 (PRC2) that mediates trimethylation of histone 3 lysine 27 [54]. Surprisingly, in our analysis, EZH2 expression did not correlate with increased trimethylation of H3K27. H3K27me3 staining was similar across all tumor grades and subgroups. This finding may point to a PRC2-independent function of EZH2 in NEN that merits further investigation. This hypothesis was further supported by the analysis of the other PRC2 core member proteins SUZ12 and EED, which were almost undetectable in all types of NEN. PRC2-independent functions of EZH2 have been described in many other tumor entities by different authors. These functions include PRC2-independent methylation or direct binding of proteins [19,25,26,27,28,29,30,55]. One example is the activation of STAT3 via methylation by phosphorylated EZH2 [28].

## 5. Conclusions

In summary, our data clearly demonstrate that EZH2 expression is associated with high-grade NENs, can be used to discriminate NET G3 from NEC, and strongly predicts the survival of patients with NEN G3. Furthermore, EZH2 might be an attractive new therapeutic target in NEN, as inhibitors of EZH2 are already available and are currently being tested in various clinical studies. Further preclinical and clinical studies will be necessary to understand the role of EZH2 in NEN fully and to evaluate the clinical benefit of EZH2 inhibitors in patients with aggressive NEN.

## Figures and Tables

**Table 1:** Characteristics of enrolled patients. GEP: gastroenteropancreatic, NEN: neuroendocrine neoplasia, P: pulmonary, UICC: Union international contre le cancer.

Characteristic	Total (n = 219)	GEP-NEN (n = 126)	P-NEN (n = 93)	*p*-Value
Age median (range) (years)	64 (18–87)	65 (18–87)	62 (20–81)	
Age No. (%) (years)				
<65	115	62 (49.2)	53 (57.0)	0.254
≥65	104	64 (50.8)	40 (43.0)	
Gender No. (%)				
Female	106	49 (38.9)	57 (61.3)	0.001 *
Male	113	77 (61.1)	36 (38.7)	
pT No. (%)				
1+2	127	50 (54.9)	77 (89.5)	<0.001 *
3+4	50	41 (45.1)	9 (10.5)	
pN No. (%)				
0	85	27 (40.9)	58 (77.3)	<0.001 *
+	56	39 (59.1)	17 (22.7)	
G No. (%)				
1+2	182	109 (86.5)	73 (78.5)	0.118
3	37	17 (13.5)	20 (21.5)	
UICC stage No. (%)				
I+II	130	53 (58.2)	77 (89.5)	<0.001 *
III+IV	47	38 (41.8)	9 (10.5)	

* = significant difference.

**Table 2:** Characteristics of enrolled patients depending on EZH2 expression. GEP: gastroenteropancreatic, NEN: neuroendocrine neoplasia, P: pulmonary, UICC: Union international contre le cancer.

Characteristic	EZH2 Low H-Score < 100 (n = 195)	EZH2 High H-Score > 100 (n = 24)	*p*-Value
Age median (range) (years)	63 (18–87)	68.5 (42–84)	
Age No. (%) (years)			
<65	105 (53.8)	10 (41.7)	0.260
≥65	90 (46.2)	14 (58.3)	
Gender No. (%)			
Female	99 (50.8)	7 (29.2)	0.046 *
Male	96 (49.2)	17 (70.8)	
Localization No. (%)			
P-NEN	82 (42.1)	11 (45.8)	
GEP-NEN	113 (57.9)	13 (54.2)	0.724
-intestinal	91 (46.7)	11 (45.8)	
-pancreatic	22 (11.3)	2 (8.3)	
pT No. (%)			
1+2	117 (75.5)	10 (45.5)	0.003 *
3+4	38 (24.5)	12 (54.5)	
pN No. (%)			
0	74 (61.7)	11 (52.4)	0.422
+	46 (38.3)	10 (47.6)	
G No. (%)			
1+2	182 (93.3)	0 (0.0)	<0.001 *
3	13 (6.7)	24 (100.0)	
UICC stage No. (%)			
I+II	118 (76.1)	12 (54.5)	0.032 *
III+IV	37 (23.9)	10 (45.5)	

* = significant difference.

**Table 3:** Multivariate analysis of EZH2-associated characteristics. UICC: Union international contre le cancer.

Characteristic	Regression Coefficient B	Standardized Beta Coefficient	*p*-Value	95% CI
Grading	77.172	0.214	0.013	3.900–32.215
pT status	18.058	0.745	0.000	64.938–89.406
UICC stage	−21.549	−0.256	0.003	−35.758–−7.341

## Data Availability

Data are contained within the supplementary material.

## Supplementary Materials

The following supporting information can be downloaded at: https://www.mdpi.com/article/10.3390/cancers14122828/s1, Table S1: patient characteristics of NEN G1/2, Table S2: patient characteristics of NEN G3, Table S3: Characteristics of enrolled patients depending on tumor localization. Figure S1: Survival in GEP-NEN and P-NEN, Figure S2: Survival in GEP-NEN and P-NEN depending on EZH2 and H3K27me3, Figure S3: Analyses depending on tumor localization.
