# Supplementary material for: Enhancer of Zeste Homolog 2 (EZH2) Is a Marker of High-Grade Neuroendocrine Neoplasia in Gastroenteropancreatic and Pulmonary Tract and Predicts Poor Prognosis
Source: Cancers (Basel). 2022 Jun 8;14(12):2828. doi: 10.3390/cancers14122828 (PMC9221317; doi:10.3390/cancers14122828)
Supplement: Supplementary file 1 [file cancers-14-02828-s001.zip › Figure S1.pdf]

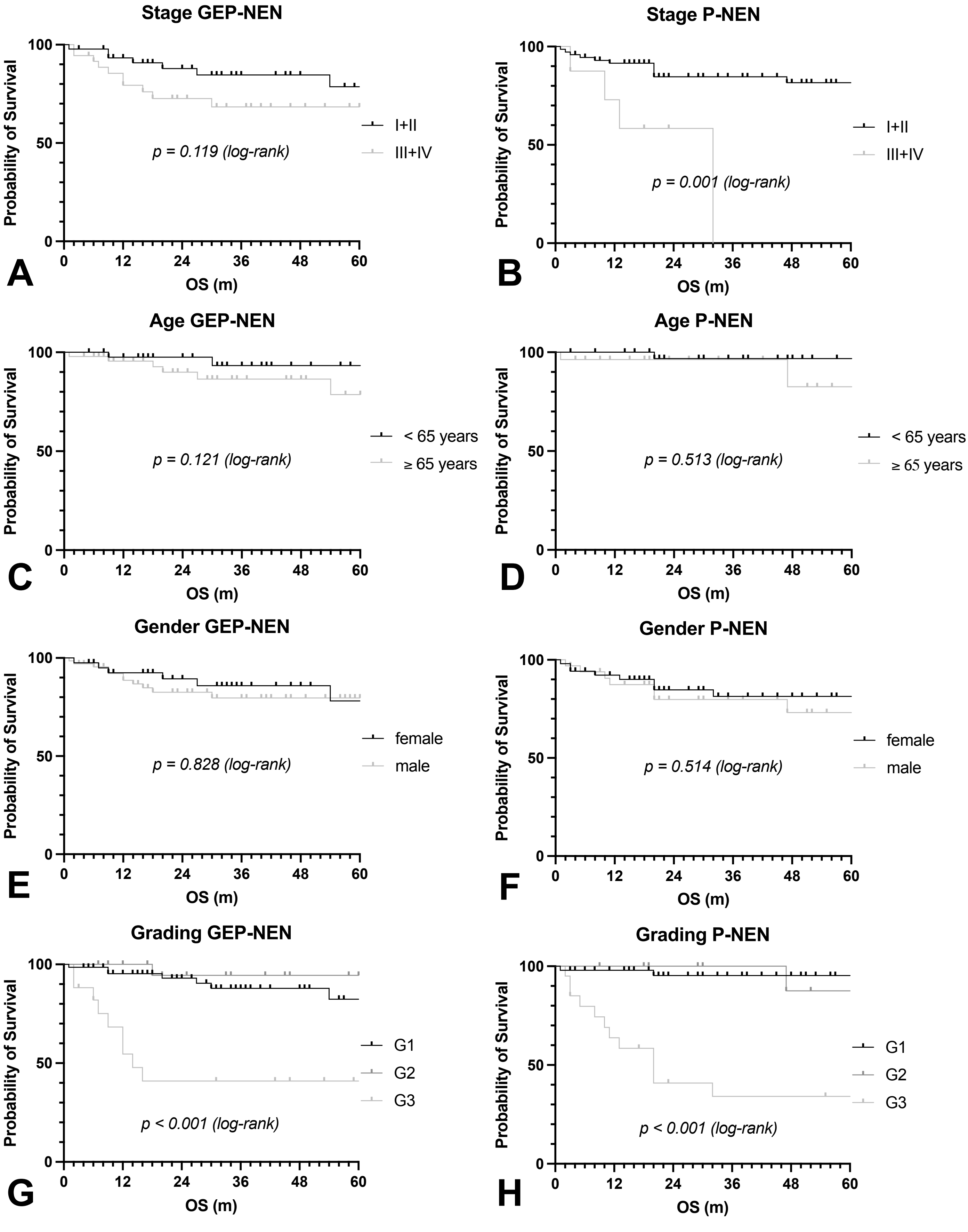
Stage GEP-NEN
Probability of Survival
p = 0.119 (log-rank)
I+II
III+IV
OS (m)
A
Stage P-NEN
p = 0.001 (log-rank)
B
Age GEP-NEN
p = 0.121 (log-rank)
< 65 years
≥ 65 years
C
Age P-NEN
p = 0.513 (log-rank)
D
Gender GEP-NEN
p = 0.828 (log-rank)
female
male
E
Gender P-NEN
p = 0.514 (log-rank)
F
Grading GEP-NEN
G1
G2
G3
p < 0.001 (log-rank)
G
Grading P-NEN
p < 0.001 (log-rank)
H
