# Supplementary material for: Enhancer of Zeste Homolog 2 (EZH2) Is a Marker of High-Grade Neuroendocrine Neoplasia in Gastroenteropancreatic and Pulmonary Tract and Predicts Poor Prognosis
Source: Cancers (Basel). 2022 Jun 8;14(12):2828. doi: 10.3390/cancers14122828 (PMC9221317; doi:10.3390/cancers14122828)
Supplement: Supplementary file 1 [file cancers-14-02828-s001.zip › Supplementary Table S3.pdf]

Supplementary Table S3. Characteristics of enrolled patients depending on tumor localization. NEN: neuroendocrine neoplasia, P: pulmonary, UICC: Union international contre le cancer

Characteristic	Intestinal NEN (n = 102)	Pancreatic NEN (n = 24)	P-NEN (n = 93)
Age median (range) [years]	65 (18-87)	63 (18-80)	62 (20-81)
Age No. (%) [years]			
< 65	50	12	53
≥ 65	52	12	40
Gender No. (%)			
Female	33	9	57
Male	62	15	36
pT No. (%)			
1+2	41	9	77
3+4	31	10	9
pN No. (%)			
0	18	9	58
+	29	10	17
G No. (%)			
1+2	89	20	73
3	13	4	20
UICC stage No. (%)			
I+II	39	14	77
III+IV	33	5	9
