# Supplementary figures and images for: Enhancer of Zeste Homolog 2 (EZH2) Is a Marker of High-Grade Neuroendocrine Neoplasia in Gastroenteropancreatic and Pulmonary Tract and Predicts Poor Prognosis
Source: Cancers (Basel). 2022 Jun 8;14(12):2828. doi: 10.3390/cancers14122828 (PMC9221317; doi:10.3390/cancers14122828)

**G3 GEP-NEN**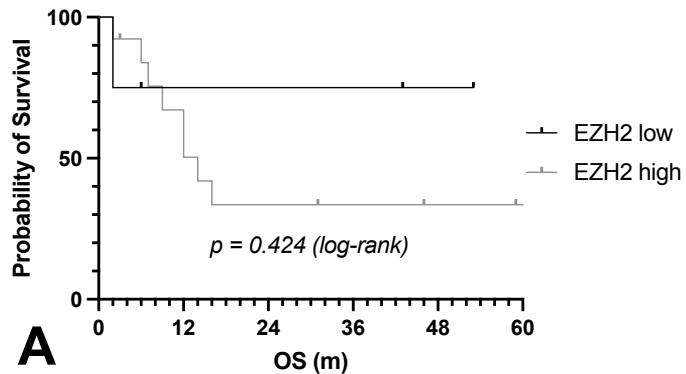**G3 P-NEN**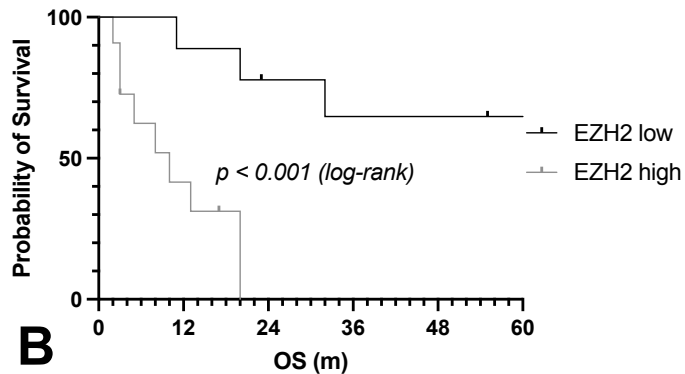**G3 GEP-NEN**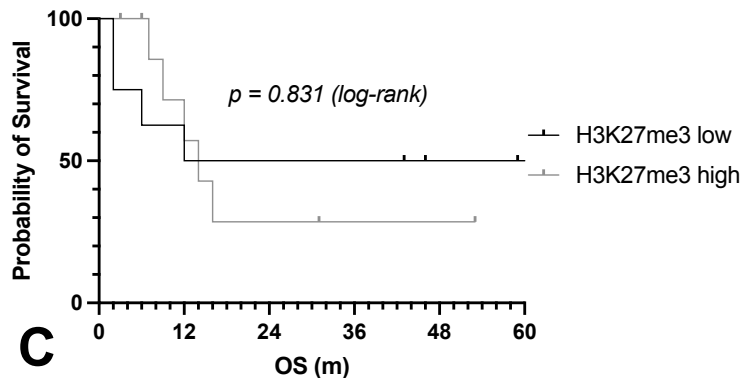**G3 P-NEN**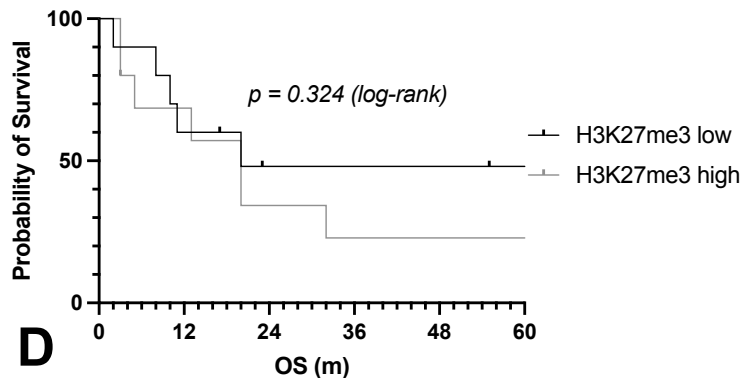

Supplement: Supplementary file 1 [file cancers-14-02828-s001.zip › Figure S2.pdf]

**G3 localization**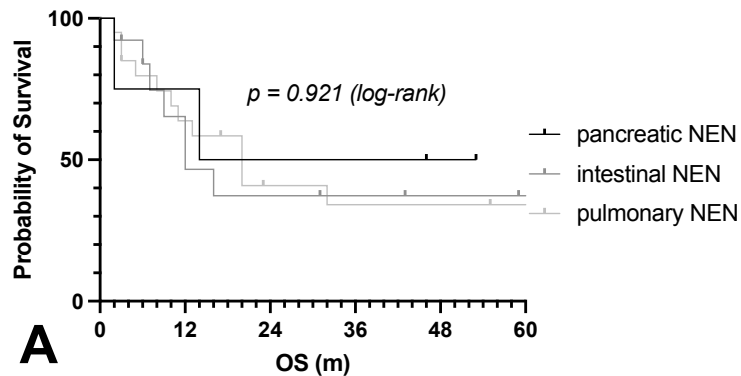**overall localization**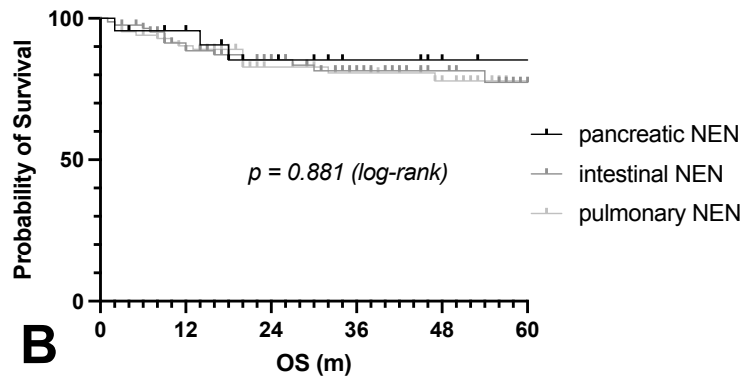**localization**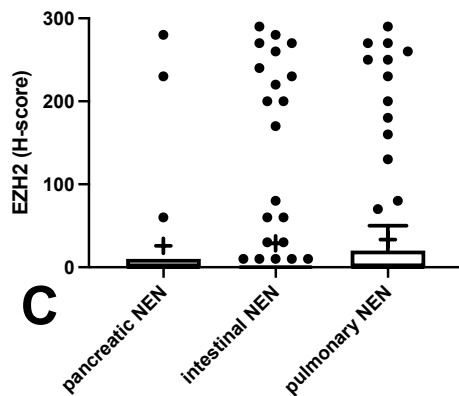**localization and grading**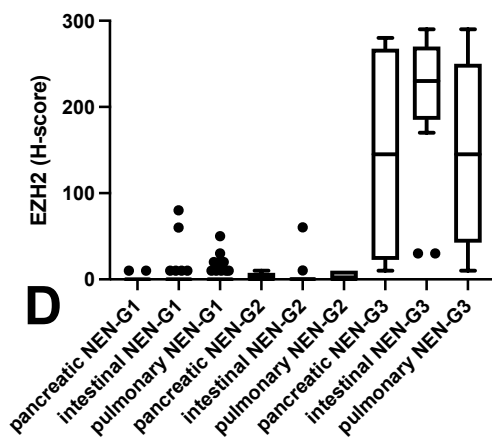

Supplement: Supplementary file 1 [file cancers-14-02828-s001.zip › Figure S3.pdf]
